# Loss-tolerant state engineering for quantum-enhanced metrology via the reverse Hong–Ou–Mandel effect

**DOI:** 10.1038/ncomms11925

**Published:** 2016-06-21

**Authors:** Alexander E. Ulanov, Ilya A. Fedorov, Demid Sychev, Philippe Grangier, A. I. Lvovsky

**Affiliations:** 1 Russian Quantum Center, 100 Novaya Street, Skolkovo, Moscow 143025, Russia; 2 Moscow Institute of Physics and Technology, Dolgoprudny 141700, Russia; 3 P.N. Lebedev Physics Institute, Leninskiy prospect 53, Moscow 119991, Russia; 4 Laboratoire Charles Fabry, Institut d'Optique Graduate School, CNRS, Université Paris-Saclay, Palaiseau 91127, France; 5 Institute for Quantum Science and Technology, University of Calgary, Calgary, Alberta, Canada T2N 1N4

## Abstract

Highly entangled quantum states, shared by remote parties, are vital for quantum communications and metrology. Particularly promising are the N00N states—entangled *N*-photon wavepackets delocalized between two different locations—which outperform coherent states in measurement sensitivity. However, these states are notoriously vulnerable to losses, making them difficult to both share them between remote locations and recombine in order to exploit interference effects. Here we address this challenge by utilizing the reverse Hong–Ou–Mandel effect to prepare a high-fidelity two-photon N00N state shared between two parties connected by a lossy optical medium. We measure the prepared state by two-mode homodyne tomography, thereby demonstrating that the enhanced phase sensitivity can be exploited without recombining the two parts of the N00N state. Finally, we demonstrate the application of our method to remotely prepare superpositions of coherent states, known as Schrödinger's cat states.

## Abstract (web summary)

N00N states are promising for quantum communications and metrology, but are vulnerable to losses. Here the authors develop a technique for preparing high-fidelity two-photon N00N states in a loss-free fashion, and demonstrate enhanced phase sensitivity without requiring recombination.

## Graphical abstract

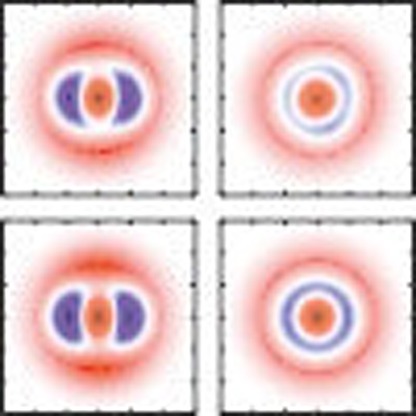

In the current race towards the practical implementation of quantum techniques for information processing and communications, a strong trend is to design loss-tolerant quantum protocols, such as the preparation of non-local superpositions of quasi-classical light states^1^, discord-assisted remote state preparation^2^, quantum illumination^3^, undoing the effect of losses on continuous-variable entanglement^4^ and the preparation of single-qubit entangled states over a long distance^5^,^6^,^7^,^8^.

In this article we are interested in N00N states 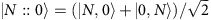, which are useful in linear-optical quantum computation^9^,^10^, quantum-optical state engineering^11^,^12^ and the preparation of photon-number path entanglement^13^,^14^. But the most important potential application of these states is as a resource for quantum enhanced metrology^14^,^15^,^16^,^17^,^18^,^19^. Interference measurements with N00N states exhibit super-resolving properties: the number of fringes per wavelength equals *N*, in contrast to a single fringe in the case of coherent states. This property can be exploited for precise measurement of diverse physical quantities. Widespread application of N00N states for metrology is however precluded by their extreme sensitivity to losses. When exposed to even moderate losses, the degree of entanglement and hence the super-resolution potential of the N00N states dramatically degrade to an extent that eliminates any advantage^20^,^21^.

In the present work, we address this challenge by developing a technique to losslessly produce N00N states between parties that are separated by a lossy quantum channel. In addition, using N00N states usually requires bringing back together the two entangled parts, introducing more propagation losses. But we will show that this second step is not required, and that super-resolution for the optical phase can be obtained remotely, by using homodyne detection^11^.

## Results

### Concept

For N00N state production, we exploit some peculiar properties of the Hong–Ou–Mandel (HOM) effect^22^, a well-known quantum interference phenomenon in which two indistinguishable photons that are overlapped on a symmetric beam splitter (BS) always emerge in the same output mode, preparing the N00N state


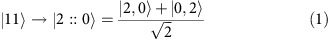


in the beam splitter output. Our experiment relies upon the reverse version of the HOM effect^23^, in which the measurements in the two output modes of the BS project them onto single-photon states. Because of the time-reversible nature of quantum mechanics, such projection is equivalent to projecting the state of the input onto the two-photon N00N state (1). If each of the beam splitter inputs is, in turn, entangled with other modes, these modes become entangled with each other, thanks to entanglement swapping^24^. Whereas the original HOM setting creates a two-photon N00N state, extension to any even *N* is straightforward (see Supplementary Section ‘Preparation of high-order N00N states').

**Figure 1:**
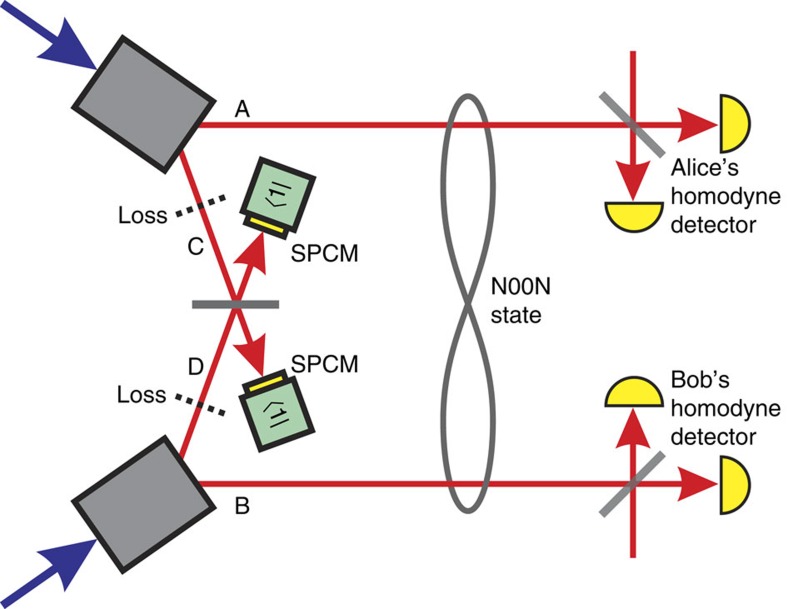
Conceptual scheme of the experiment. A coincidence click of the SPCMs projects modes C and D onto the two-photon N00N state due to the reverse HOM effect. This, in turn, prepares modes A and B in the same state, thanks to entanglement swapping.

Specifically, consider a set-up in which two pairs of modes (A,C) and (B,D) are prepared in two-mode squeezed states:


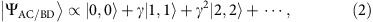


by means of non-degenerate parametric down-conversion. Modes C and D are then mixed on a symmetric BS, the outputs of which are subjected to measurement in the photon-number basis via single-photon counting modules (SPCMs), as shown in Fig. 1.

In the weak-squeezing limit |*γ*|^2^<<1, every SPCM click is likely to be caused by no more than one photon. Then, a coincidence click in both SPCMs correspond to projection on state |1, 1〉_CD_. Due to the unitary nature of the BS operation, this event assures that modes C and D were initially in the N00N state (1). This corresponds to two pairs of photons having been produced in either of two crystals; therefore, now the remaining modes A and B also share a two-photon N00N state. The application of the reverse HOM effect to N00N state preparation has been proposed by Kok *et al*.^13^, albeit not in a remote fashion as we do here.

A remarkable feature of this scheme is its robustness to losses in channels C and D. Such losses only reduce the probability of the two SPCMs to click, but, if the clicks do occur and the down-conversion amplitude *γ* is sufficiently small, the leading term in the state of channels A and B is still the two-photon N00N state. We show in Supplementary equation (8) and Supplementary Fig. 4 that the fidelity of this state is bounded from below by *F*⩾1−4*γ*^2^ for all loss values. Therefore, by pumping the down-conversion crystals sufficiently weakly, one can prepare a N00N state of arbitrarily high purity, no matter how high the losses. Our method is equally robust with respect to low quantum efficiencies of the SPCMs, because these are effectively equivalent to additional losses in modes C and D^25^. Note, however, that our technique does not help eliminate losses in modes A and B, as well as the effect of detector imperfection in these modes.

### N00N state tomography

**Figure 2:**
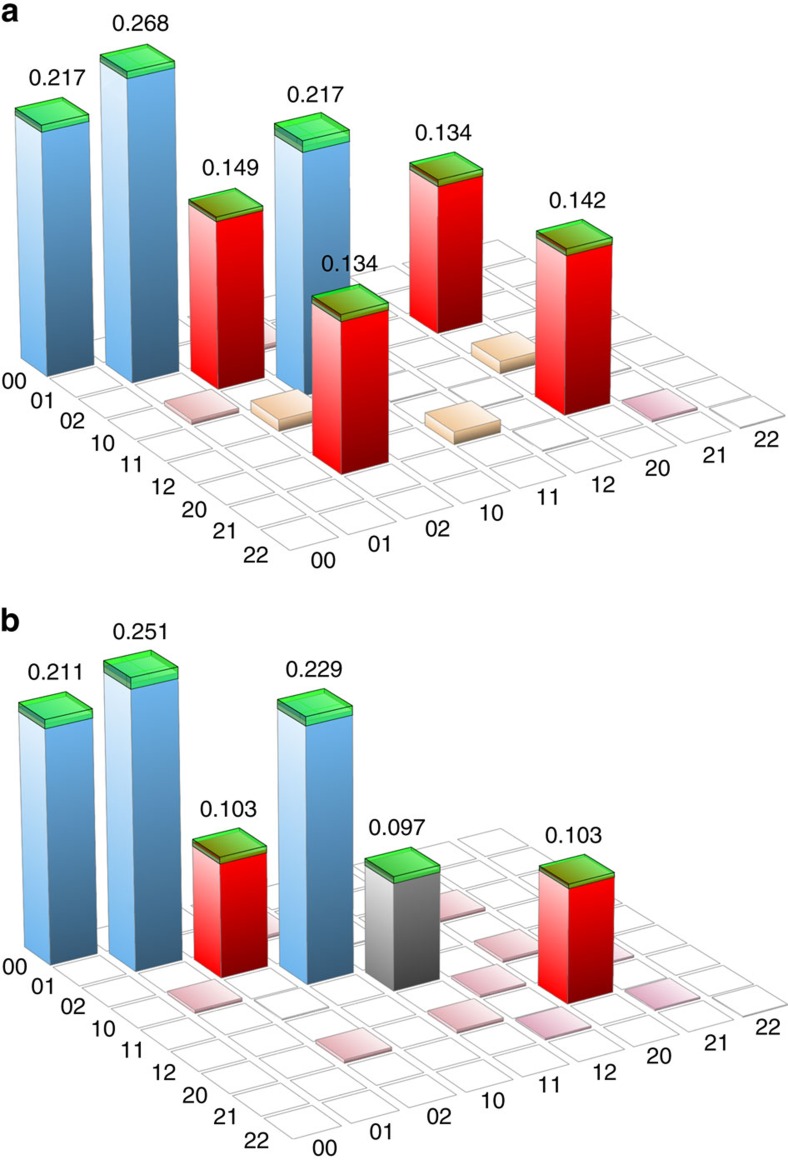
Density matrix of the conditionally prepared state of modes A and B. The data are displayed with a 10-dB total loss in modes C and D before the BS, reconstructed in the Fock basis. No correction is made for losses in any of the channels. The numbers along the horizontal axes are the indices of the bra and ket elements of that matrix. The components shown in red correspond to the ideal N00N state, others appear due to losses. (**a**) When modes C and D are matched to each other, a two-photon N00N state is prepared. The |1, 1〉〈1, 1| component amounts to 0.01. (**b**) In the case of a 5-ps mismatch between modes C and D, the HOM interference and any coherence between modes A and B are eliminated. The emerged |1, 1〉〈1, 1| component is shown in grey. Green hats show the statistical error of the reconstruction.

Conditioned on coincidence clicks of the two SPCMs, we characterize the state of modes A and B by means of homodyne tomography (Methods section). The measured states in both cases are very close to the ideal state (1) that has suffered a 1–*η*=45% loss due to imperfect detection efficiency^26^,^27^ (Fig. 2a).

**Figure 3:**
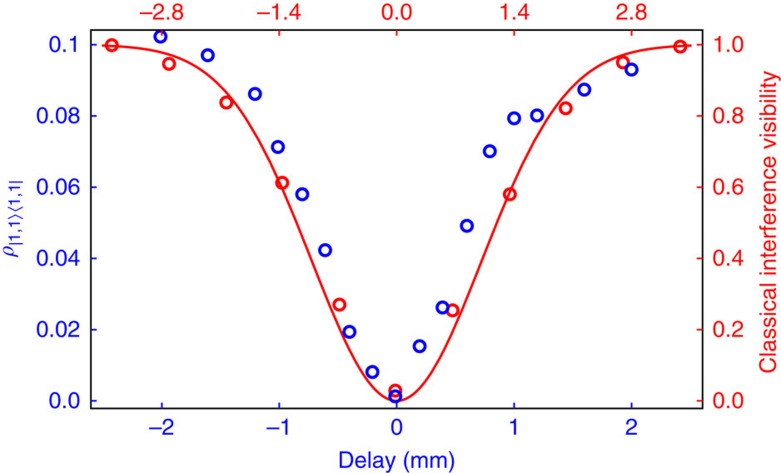
The reverse HOM dip. Quantum and classical interference as a function of the delay between modes C and D. The blue points represent the weight of the |1, 1〉〈1, 1| component in the reconstructed density matrix (obtained from homodyne tomography, not from photon counting). The red points and red curve correspond to the visibility of the classical interference between the master laser pulses that underwent the same spectral filtering as quantum modes C and D. The horizontal axes for the classical and quantum cases are scaled by a factor of 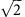 with respect to each other to match the theoretically expected widths of the interference patterns.

To illustrate the reverse HOM effect and the critical significance of the matching between modes C and D for proper N00N state preparation, we measured the behaviour of the state in modes A and B as a function of increasing temporal mismatch between modes C and D. As evidenced by Fig. 3, the fraction of the biphoton component |1,1〉 in that state exhibits a dip that is characteristic as a signature of the HOM effect. The tomographic state reconstruction for the case of complete mismatch is shown in Fig. 2b. In addition to a macroscopic biphoton component, no off-diagonal terms are present in this case because no coherence between modes A and B can emerge in the absence of interference.

### Phase sensitivity enhancement

The enhanced phase sensitivity is manifested by the mean values of observables *X*_A_*X*_B_ and 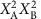, where *X*_A_=*Q*_A_ cos *θ*_A_+*P*_A_ sin *θ*_A_ and *X*_B_=*Q*_B_ cos *θ*_B_+*P*_B_ sin *θ*_B_, are quadrature operators of modes A and B. For the one-photon 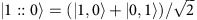 and two-photon N00N states one has, respectively,


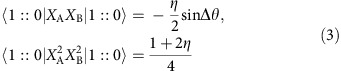


and


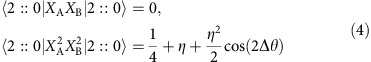


where Δ*θ*=*θ*_A_−*θ*_B_ and we assumed the phases in modes C and D to be constant.

We see that in order to compare between the one- and two-photon N00N states using quadrature measurements, we need to use different observables. This notwithstanding, the phase dependence of the appropriate observable in each state is as expected, that is, with period 2*π*/*N* for each *N*-photon N00N state.

**Figure 4:**
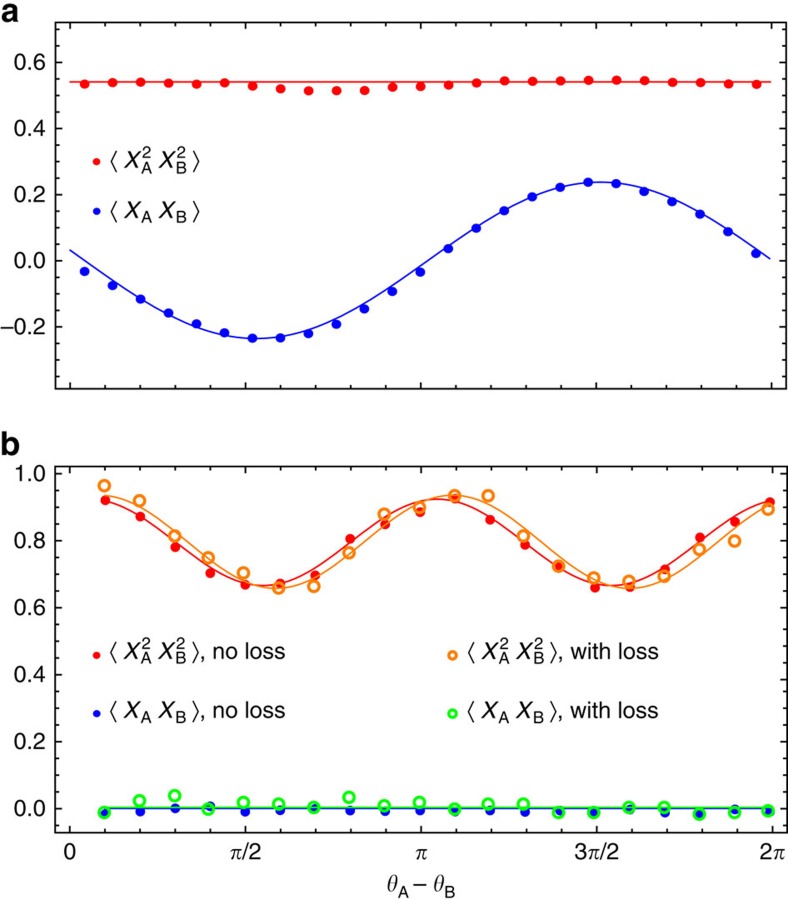
Periodic phase-dependent behaviour of N00N states. Dependence of the mean and mean-square product of Alice's and Bob's quadratures on the difference *θ*_A_−*θ*_B_ of Alice's and Bob's phases for states |1::0〉 (**a**) and |2::0〉 (**b**) is shown. Enhanced phase sensitivity is evident for the two-photon N00N state. Solid lines are theoretical predictions.

An experimental check of this behaviour is demonstrated in Fig. 4. In agreement with the results of state reconstruction, the second-order interference of the |2 :: 0〉 state, generated in the presence of the loss, exhibits the same visibility as without loss.

### Loss-tolerant preparation of Schrödinger's cat states

Being path-entangled, the N00N state can also be used for single-mode quantum-state engineering. Associating modes A and B with fictitious observers Alice and Bob, we consider a setting in which Alice, by performing quadrature measurements on her mode, remotely prepares a state in Bob's mode. Neglecting inefficiencies, Alice's quadrature outcome *X* measured at phase angle *θ* brings the Bob's mode to the state


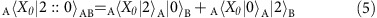


where


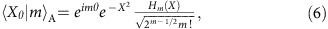


are Fock state wavefunctions, with *H*_*m*_(*X*) being Hermite polynomials. In this way, by postselecting specific values of Alice's observed quadrature, one can generate arbitrary superpositions of the 0- and 2-photon states, which approximate the even coherent-state superpositions (CSS) |*α*〉+|−*α*〉, sometimes viewed as a quantum-optical implementation of the Schrödinger's cat paradox^11^,^12^,^28^,^29^.

**Figure 5:**
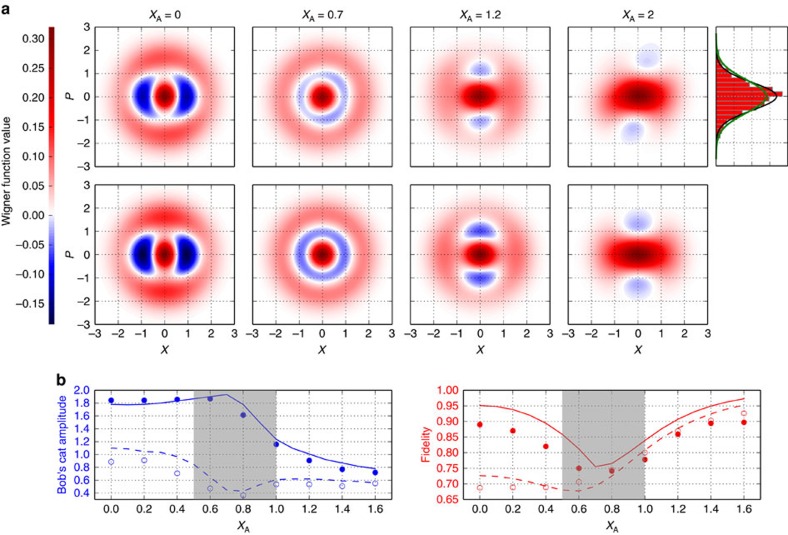
Schrödinger's cat states. (**a**) Bob's Wigner functions after conditioning on Alice's quadrature measurement, with 55% efficiency correction in Bob's mode. Top row: experimental results. Bottom row: theoretical simulation. Fidelities between experiment and theory are 0.98, 0.98, 0.97, 0.99 from left to right. The top-right panel shows the marginal distribution of Bob's position quadrature in the case of *X*_A_=2, which exhibits squeezing. The black line fits the data, the green line corresponds to the vacuum state. (**b**) Amplitude of the CSS that best approximates the state of Bob's mode versus Alice's quadrature, and the corresponding fidelity. Lines show theoretical predictions. Filled (empty) markers and solid (dashed) lines stand for the data with (without) the efficiency correction. The approximation of Bob's state with a CSS state near *X*=0.7 (second column) is unreliable because of the unproportionally large two-photon fraction and low phase dependence; this instability causes opposite behaviour of the blue lines in the shaded region.

The states of Bob's mode for different outcomes of Alice's homodyne measurement are displayed in Fig. 5. Projection on *X*=0 (Fig. 5, first column) results in superposition 0.052|0〉−0.85|2〉, partially mixed due to losses in Alice's channel. After correcting for Bob's homodyne detection inefficiency, this state has a fidelity of 0.88 with the even CSS state of amplitude *α*=1.84, squeezed with parameter *z*=0.48. This value compares favourably with state-of-the-art results^12^,^29^, with the added advantage that our protocol is tolerant to the losses in the optical channel between Alice and Bob.

Effective CSS amplitudes and approximation fidelities for other values of *X* are shown in Fig. 5b. With increasing *X*, the two-photon fraction initially increases relative to the vacuum because the two-photon state wavefunction 〈*X*|2〉 decreases faster than the vacuum wavefunction 〈*X*|0〉. Wavefunction 〈*X*|2〉 changes sign near the value *X*=0.7, where ideally a pure two-photon state in Bob's mode should be observed. In practice, due to the losses and finite width of Alice's post-selection window, a phase-insensitive mixture of 0.4|0〉〈0|+0.6|2〉〈2| is produced (second column). In this region, the remotely prepared state approximates the CSS poorly because of the high two-photon component. For higher values of X, this two-photon component in Bob's state reduces again, resulting in increasingly faithful approximation of even CSSs with decreasing amplitudes (third column). For very low amplitudes, this state approximates a weakly squeezed vacuum state. This is the case for *X*=2 (fourth column): Bob's quadrature spectrum exhibits squeezing by 0.65±0.24 dB (without efficiency correction). The corresponding quadrature histogram is shown in the upper right panel of Fig. 5.

## Discussion

The protocol developed here addresses the primary challenge in the way of employing nonclassical states of light, particularly the N00N state, for quantum metrology: optical losses. With conventional optical fibres, it allows establishing nearly ideal N00N entanglement over large distances, with a considerably increased tolerance to the loss in the channel connecting the parties. Due to its extreme sensitivity to the overall phase between Alice and Bob (see Supplementary Section ‘Phase behavior' for details), our scheme could be used for ultra-precise measurements of the distance between Alice's and Bob's homodyne detectors. Its further advantage is that there is no need to recombine the two parts of the N00N states, thanks to the homodyne detections.

In many cases, however, it is likely that a simple laser interferometric measurement would do better than our scheme due to the much larger number of available photons. Our scheme becomes advantageous in settings where the amount of light transmitted between the two parties is limited. Suppose, for instance, that a lossy biological medium, very sensitive to the light intensity, is inserted in channels C and/or D. Then it will be possible to get high-precision interferometric measurements through this medium, with almost negligible light intensity, as long as channels A, B and the associated homodyne detections have a high quality.

At this stage our experiment is a proof-of-principle only, and its application in practical settings would involve a number of challenges—in particular, the synchronization of the photons in channels C and D within the inverse down-conversion bandwidth. Furthermore, the production of higher-order N00N states would be required to achieve a significant practical gain over interferometry with coherent states, which are not limited in photon number. Our scheme can be generalized to enable producing N00N states with an arbitrary even *N*. To this end, we can use a sequence of *N*/2 reverse HOM measurements to subject modes C and D to operator 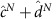 using the recipe of Kok *et al*.^13^. An event in which the application of this operator has been successful (all *N* SPCMs have clicked) implies that either C or D initially contained at least *N* photons, but is unable to distinguish which one. This means, in turn, that modes A and B are in the |*N *:: 0〉 state. Similarly to the two-photon case, the down-conversion amplitude *γ* must be sufficiently low in order to reduce the contribution of higher-order terms in the output state. We present a detailed theoretical and numerical analysis of this scheme in the Supplementary Section ‘Preparation of high-order N00N states'. In particular, we find that the fidelity of the prepared state for a given *γ* does not strongly depend on the losses in modes C and D. This enables preparation of high N00N states at reasonable rates, even with quite lossy channels.

In addition to interferometry, the generalization of our scheme to higher-order N00N states may have other applications, based on ultraremote preparation of quantum states of light, as illustrated by the ‘cat state' generation presented above. In that case, fibre transmission over long distances may be used, with possible quantum cryptography applications. The present scheme is therefore a valuable addition to the quantum-state engineering toolbox, for both discrete- and continuous-variable degrees of freedom of optical modes^11^,^30^,^31^.

## Methods

### Experimental details

We employ a pulsed Ti:Sapphire laser (Coherent Mira 900D) with a wavelength of 780 nm, mean power 1.3 W, repetition rate of 76 MHz and a pulse width of ∼1.6 ps. Most of the laser output is directed into an lithium triborate crystal for frequency doubling. We obtain up to 300 mW second harmonic; after subsequent spectral cleaning, about 100 mW remain. Then we implement parametric down-conversion in two periodically poled potassium titanyl phosphate crystals in a type II spectrally and spatially degenerate, but polarization non-degenerate configuration. The single-photon detection is implemented using SPCMs by Excelitas. The modes entering the SPCMs are spectrally filtered and delivered to the detectors by means of single-mode fibres, which ensures proper preparation of the heralded photon mode^26^,^27^. Including the filters and fibres, the quantum efficiency of these detectors is estimated as *η*_SPCM_∼0.15.

Without losses, the experimental double-coincidence event rate is ∼100 Hz, which corresponds to a probability of ∼10^−6^ per pulse and the down-conversion amplitude *γ*^2^∼0.007. With a total of 10 dB loss equally distributed between modes C and D, the coincidence rate decreases by a factor of 10 due to the reduction of the equivalent SPCM efficiency down to ∼0.1.

A total of 500,000 quadrature samples have been acquired in each setting for the tomographic reconstruction of the N00N state. The density matrix was obtained from these data using the iterative maximum likelihood algorithm^32^,^33^,^34^, which in case of N00N states requires the knowledge of the phase difference between two local oscillators at each moment in time. This information was collected from the quadrature correlations exhibited by |1 :: 0〉 state (Fig. 4, top), which was produced upon a single click of one of the SPCMs. The rate of these events was about 100 kHz, which allowed us to evaluate the quadrature correlation and hence the phase at any moment in time with a sufficient precision.

### Data availability

The data that support the findings of this study are available from the corresponding author upon request.

## Additional information

**How to cite this article:** Ulanov, A. E. *et al*. Loss-tolerant state engineering for quantum-enhanced metrology via the reverse Hong–Ou–Mandel effect. *Nat. Commun.* 7:11925 doi: 10.1038/ncomms11925 (2016).

## Supplementary Material

Supplementary Information Supplementary Figures 1-4, Supplementary Discussion and Supplementary References.
