# Supplementary material for: Loss-tolerant state engineering for quantum-enhanced metrology via the reverse Hong–Ou–Mandel effect
Source: Nat Commun. 2016 Jun 21;7:11925. doi: 10.1038/ncomms11925 (PMC4919515; doi:10.1038/ncomms11925)
Supplement: Supplementary Information — Supplementary Figures 1-4, Supplementary Discussion and Supplementary References. [file ncomms11925-s1.pdf]

SUPPLEMENTARY FIGURES

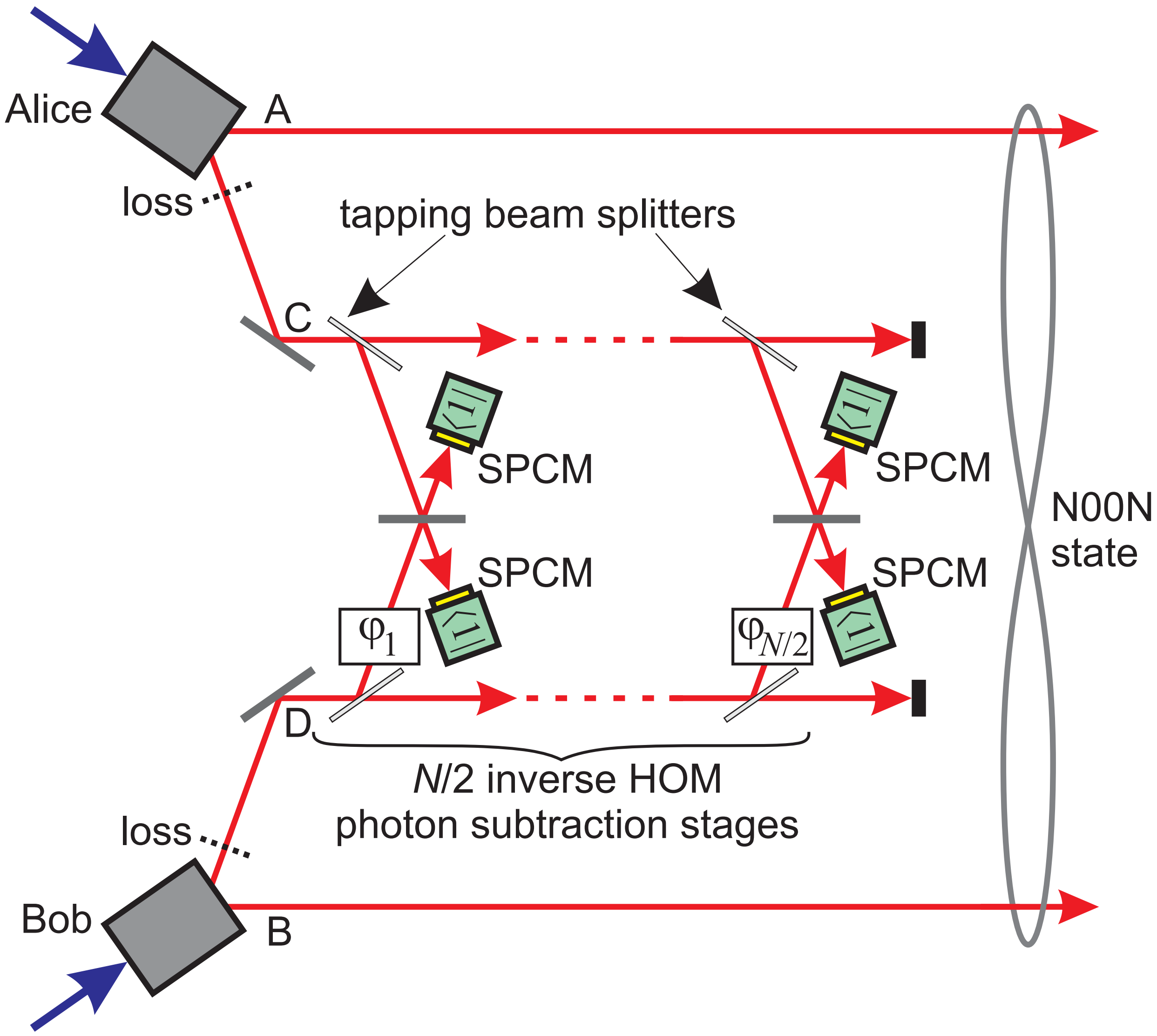

Supplementary Figure 1. Preparation of $|N :: 0\rangle$ states with arbitrary even $N \geq 2$.

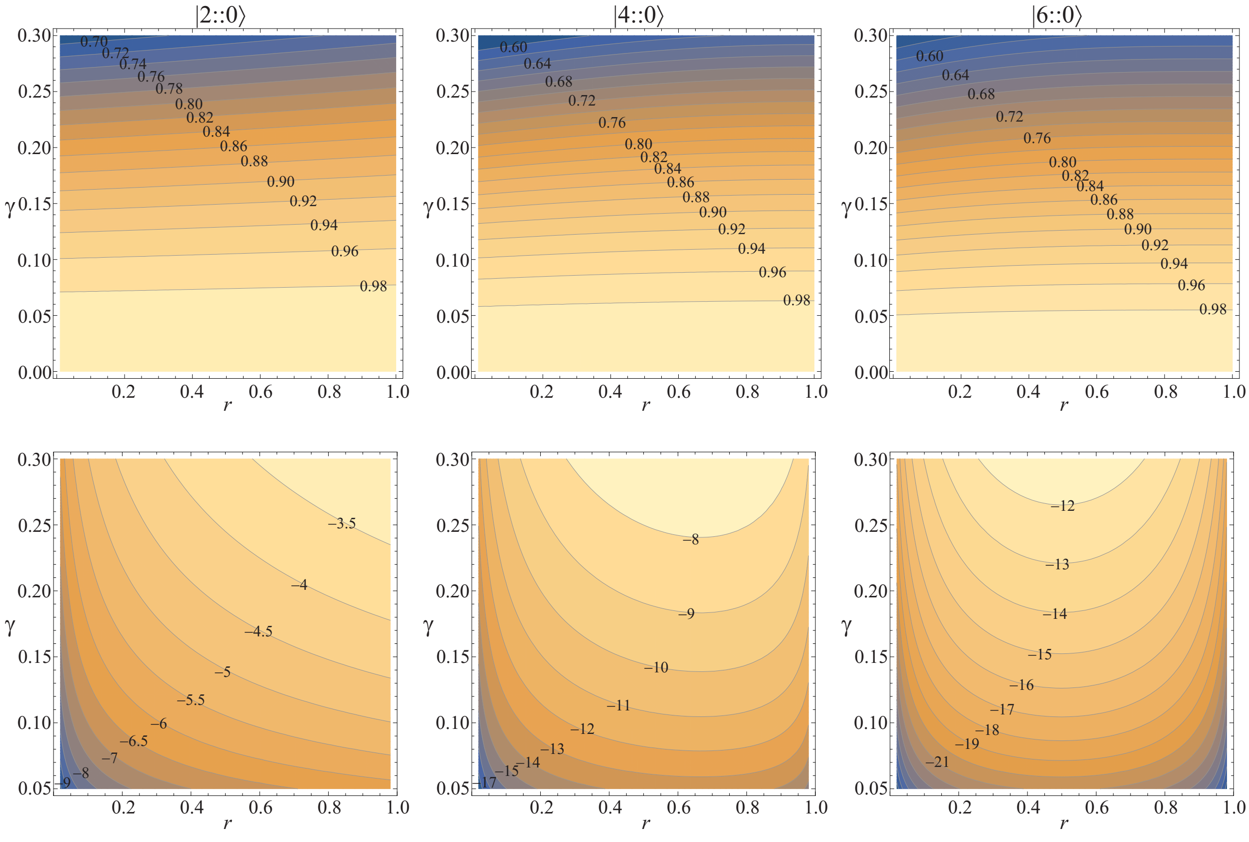

Supplementary Figure 2. Preparation fidelity (top row) and common logarithm of the preparation probability (bottom row) of the first three N00N states as functions of the squeezing parameter γ and the amplitude reflectivities $\sqrt{r}$ of the tapping BSs. The total loss in modes C and D is 10 dB and the SPCMs are non-discriminating.

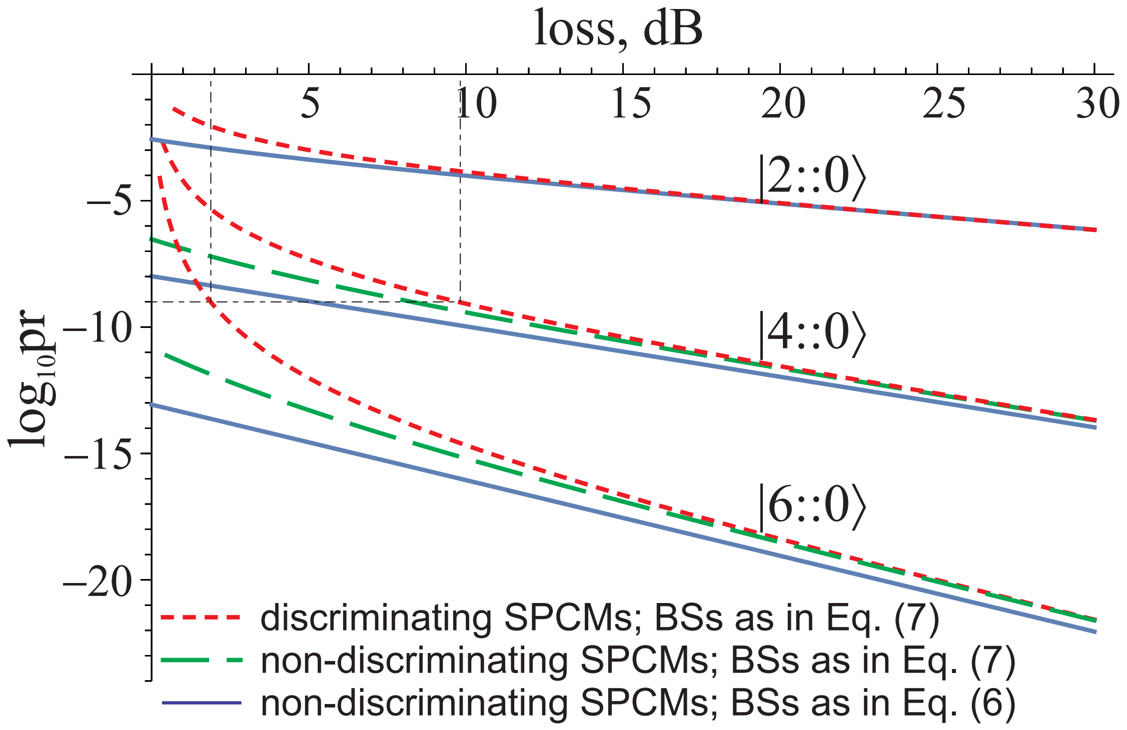

Supplementary Figure 3. Preparation probability of the first three even N00N states as a function of the total loss in modes C and D. The values of γ are chosen to provide a 90% fidelity. The dot-dashed black lines show the amount of loss corresponding to the preparation probability of 10^{-9}.

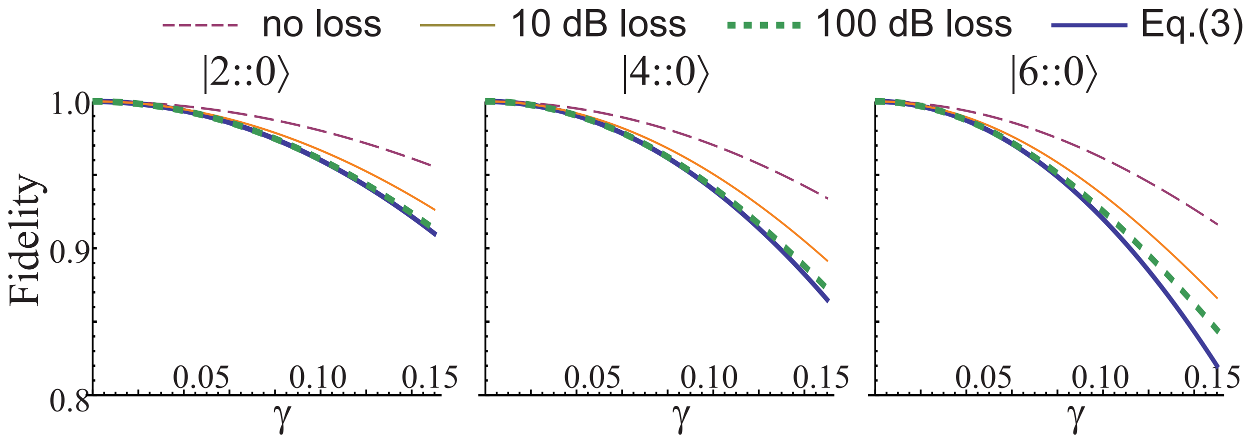

Supplementary Figure 4. Preparation fidelity of the first three even N00N states as a function of the parametric down-conversion amplitude γ under various losses and the boundary set by Eq. (8). The reflectivities of the tapping BSs are set according to Eq. (12); the SPCMs are non-discriminating.

SUPPLEMENTARY DISCUSSION

Phase behavior

In this section, we formally derive the dependence of the prepared 2-photon N00N state on the phases $\theta_{\rm A}, \theta_{\rm B}, \theta_{\rm C}, \theta_{\rm D}$, accrued as a result of propagation through channels A, B, C, D respectively (Fig. 1 in the main text). We start with the 4-mode output state of the two down-converters $|\Psi_{\rm AC}\rangle \otimes |\Psi_{\rm BD}\rangle$ [where $\left|\Psi_{\rm AC/BD}\right\rangle$ is given by Eq. (2) in the main text], expanding it to the second order in γ:

$$
\begin{aligned}
|\Psi_{\rm AC}\rangle \otimes |\Psi_{\rm BD}\rangle = {} & |0,0,0,0\rangle \\
& + \gamma(|1,1,0,0\rangle + |0,0,1,1\rangle) \\
& + \gamma^2(|2,2,0,0\rangle + |1,1,1,1\rangle + |0,0,2,2\rangle) \\
& + o(\gamma^2),
\end{aligned} \tag{1}
$$

where, throughout this Supplementary, the photon numbers in each ket refer to modes A, C, D, B, in that order. In this expression, only term $\gamma^2(|2,2,0,0\rangle + |0,0,2,2\rangle)$ can give rise to a coincidence click of the two SPCM. Indeed, the terms of the zeroth and first order in γ contain less than two photons in modes C and D and term $\gamma^2\,|1,1,1,1\rangle$ cannot bring about a coincidence event due to the Hong-Ou-Mandel (HOM) effect. We will be omitting these terms in the calculation below for brevity.

Applying the phase shift operator [1] $\hat{U}_i = e^{i\theta_i \hat{n}}$ to each mode of the above state transforms it into

$$
\begin{aligned}
& \hat{U}_{\rm A}\hat{U}_{\rm B}\hat{U}_{\rm C}\hat{U}_{\rm D}\gamma^2(|2,2,0,0\rangle + |0,0,2,2\rangle) \\
& = \gamma^2(e^{2i(\theta_{\rm A}+\theta_{\rm C})}\,|2,2,0,0\rangle + e^{2i(\theta_{\rm B}+\theta_{\rm D})}\,|0,0,2,2\rangle).
\end{aligned} \tag{2}
$$

In the two terms of the above expression, modes C and D are in states $|2,0\rangle$ and $|0,2\rangle$, respectively. Applying the symmetric beam splitter (BS) operator $\hat{B}_{CD}$ to these states leads to [1]

$$
\begin{aligned}
\hat{B}_{\rm CD}\,|2,0\rangle &= \frac{1}{2}\,|2,0\rangle + \frac{i}{\sqrt{2}}\,|1,1\rangle - \frac{1}{2}\,|0,2\rangle\,; \\
\hat{B}_{\rm CD}\,|0,2\rangle &= -\frac{1}{2}\,|2,0\rangle + \frac{i}{\sqrt{2}}\,|1,1\rangle + \frac{1}{2}\,|0,2\rangle\,.
\end{aligned} \tag{3}
$$

When, after the BS, modes C and D are projected onto $|1,1\rangle_{\rm CD}$ through the photon number

measurement, the state of modes A and B becomes

$$\begin{aligned}&\langle 1,1|_{\mathrm{CD}}\,\hat{B}_{\mathrm{BD}}\hat{U}_{\mathrm{A}}\hat{U}_{\mathrm{B}}\hat{U}_{\mathrm{C}}\hat{U}_{\mathrm{D}}\gamma^2(|2,2,0,0\rangle+|0,0,2,2\rangle)\\&=\frac{i}{\sqrt{2}}\gamma^2(e^{2i(\theta_{\mathrm{A}}+\theta_{\mathrm{C}})}\,|2,0\rangle+e^{2i(\theta_{\mathrm{B}}+\theta_{\mathrm{D}})}\,|0,2\rangle)_{\mathrm{AB}}).\end{aligned} \tag{4}$$

We see that the resulting N00N state features doubly enhanced sensitivity to the phases in all four channels.

Preparation of high-order N00N states

In the main text, we have shown that projecting modes C and D from Alice's and Bob's parametric amplifiers onto the two-photon N00N state collapses the counterpart pair of modes, A and B, onto the same state. Here we discuss a generalization of our scheme, capable of producing the N00N state with an arbitrary even N.

In the setup shown in Supplementary Figure 1. modes C and D are subjected to operator $\hat{c}^N+\hat{d}^N$ using the recipe of Kok *et al.* [2]. This operator is implemented as a sequence of $N/2$ operators of form $\hat{c}^2+e^{i\theta_k N}\hat{d}^2$ using the decomposition

$$\hat{c}^N+\hat{d}^N=\prod_{k=1}^{N/2}(\hat{c}^2+e^{i\theta_k}\hat{d}^2), \tag{5}$$

where $\theta_k=4\pi k/N$. To realize each step of this sequence, the two modes are tapped using weakly reflective BSs. The tapped modes are mixed on a symmetric BS and detected using SPCMs. In this way, the state of the tapped modes is projected onto the two-photon N00N state thanks to the reverse HOM effect, which means that one of the modes C or D loses two photons while the other one stays unaffected. A phase shift of $\theta_k/2$ is applied to one of the tapped modes in order to account for factor $e^{i\theta_k}$ in Eq. (5).

An event in which the application of operator $\hat{c}^N+\hat{d}^N$ to modes C and D has been successful (all N SPCMs have clicked) implies that either of the two modes contained at least N photons before reaching the first BS. The lowest-order terms in $|\Psi_{\mathrm{AC}}\rangle\otimes|\Psi_{\mathrm{BD}}\rangle$ that satisfy this requirement are

$$\begin{aligned}&\gamma^N(|N,N,0,0\rangle+|0,0,N,N\rangle)\\&+\gamma^{N+1}(|N,N,1,1\rangle+|1,1,N,N\rangle\\&+|N+1,N+1,0,0\rangle+|0,0,N+1,N+1\rangle)\\&+o(\gamma^{N+1}),\end{aligned} \tag{6}$$

where we neglected the phase shifts. Applying operator $\hat{c}^N + \hat{d}^N$ to modes C and D therefore produces the following state:

$$
\begin{aligned}
&(\hat{c}^N + \hat{d}^N)\,|\Psi_{\mathrm{AC}}\rangle \otimes |\Psi_{\mathrm{BD}}\rangle \\
&= \sqrt{N!}\gamma^N(|N,0,0,0\rangle + |0,0,0,N\rangle) \\
&+ \gamma^{N+1}\sqrt{N!}(|N,0,1,1\rangle + |1,1,0,N\rangle) \\
&+ \gamma^{N+1}\sqrt{(N+1)!}(|N+1,1,0,0\rangle + |0,0,1,N+1\rangle) \\
&+ o(\gamma^{N+1}),
\end{aligned} \tag{7}
$$

When modes C and D are discarded, the first term in the right-hand side of Eq. (7) yields state $|N :: 0\rangle$ in modes A and B, while higher-order terms can be neglected for a sufficiently low γ.

We calculated the output density matrix of the scheme shown in Supplementary Figure 1 with one, two and three stages (which corresponds to states $|2 :: 0\rangle$, $|4 :: 0\rangle$, $|6 :: 0\rangle$, respectively) numerically for a variety of settings. The calculations were performed in the Fock basis truncated at the 7-photon level.

First, we analyzed the setting in which the reflectivities of all the tapping BSs for a given N were set equal to each other and the SPCMs were assumed to be not number-discriminating. The efficiency of the SPCMs was set to unity (imperfect SPCM efficiency can be treated in the same way as additional losses in channels C and D [3]). The SPCM dark counts are neglected.

Supplementary Figure 2 shows the preparation efficiency and base 10 logarithm of the preparation probabiliity of the respective N00N states, assuming that the total loss in channels C and D is 10 dB, i.e. the same as in our experiment. We find that, by choosing a sufficiently low squeezing parameter, one can theoretically achieve a fidelity that is arbitrarily close to unity. This observation is consistent with the above theoretical analysis. Indeed, the conditional preparation fidelity of the N00N state is equal to the weight of the first term in the renormalized right-hand side of Eq. (7). Neglecting terms of order $N + 2$ and higher in γ, we find that the fidelity scales for small values of γ as

$$F \approx 1 - (N + 2)\gamma^2. \tag{8}$$

This behavior is confirmed by the numerical calculations shown in the top row of Supplementary Figure 2.

Usually, a tapping BS needs to have a miniscule reflectivity in order to implement the photon annihilation with a high fidelity. This is required in order to reduce the probability of events in which the non-discriminating detector that heralds the annihilation would fire in response to more than one photon. In the present case, however, the N00N state preparation fidelity does not show strong dependence on that reflectivity (Supplementary Figure 2, top row). This is because, in order for the N00N state creation to be heralded, all N SPCMs in Supplementary Figure 1 must fire simultaneously, which means that at least N photons must be present in modes C or D when they reach the heralding station. In order for a particular SPCM to receive more than one photon, these modes would need to contain have more than N photons, the probability of which would have a lower order in γ^2.

By the same token, the preparation fidelity is not strongly dependent on the amount of loss in modes C and D. Therefore, in theory, our method can be used to prepare high-quality N00N states no matter how significant the loss between the parties.

In practice, though, a low squeezing parameter combined with high loss in the channel between Alice and Bob will drastically reduce the probability to obtain a heralded N00N state. This probability can be estimated for low γ as follows. The probability for either Alice's or Bob's squeezed vacuum state to contain term $|N, N\rangle$, which gives rise to the N00N state, is $2\gamma^{2N}$. If the transmissivity of the channel connecting Alice and Bob is τ and assuming that the heralding station is midway between Alice and Bob, the probability that a photon from either party reaches the heralding station is $\sqrt{\tau}$, and hence the probability that all of these photons reach the heralding station is $\tau^{N/2}$. Finally, in order for the N00N state to be heralded, it is required that each of these N photons are distributed to a different SPCM, leading to an additional probability factor $C(r_n)$. Hence the heralding probability is

$$\mathrm{pr} = 2C(r_n)\gamma^{2N}\tau^{N/2}, \tag{9}$$

where r_n, for $n = 1, \ldots, N/2$, denote the reflectivities of the tapping BSs. Let us calculate and optimize $C(r_n)$. When N photons, e.g. in mode C, arrive at the first tapping BS, it is required for a heralding event that 2 photons be reflected and $N-2$ transmitted. This will occur with probability $\begin{pmatrix} N \\ 2 \end{pmatrix} r_1^2(1-r_1)^{N-2}$.The two tapped photons will be separated by the central BS and land on two different SPCMs with probability 1/2. Continuing this

argument in sequence for all tapping BSs, we find

$$C(r_n) = \prod_{n=1}^{N/2} \begin{pmatrix} N-2(n-1) \\ 2 \end{pmatrix} \frac{r_n^2(1-r_n)^{N-2n}}{2} \tag{10}$$

$$= \frac{N!}{2^N} \prod_{n=1}^{N/2} r_n^2(1-r_n)^{N-2n}.$$

If all tapping BS reflectivities are the same ($r_n = r$), this expression reduces to

$$C(r_n) = \frac{N!}{2^N} r^N (1-r)^{\frac{N}{2}\left(\frac{N}{2}-1\right)}.$$

By calculating the derivative of this result with respect to r, we find that the maximum is obtained for

$$r = \frac{4}{N+2}, \tag{11}$$

which corresponds to $r = 1$, $2/3$ and $1/2$ for $N = 2$, 4 and 6, respectively. This is verified by the numerical calculations shown in Supplementary Figure 2 (bottom row). The corresponding numerically calculated N00N state heralding probabilities are displayed by solid blue lines in Supplementary Figure 3 and are in agreement with the above theoretical predictions.

It is however evident from Eq. (10) that the highest preparation probability is obtained when the tapping BS reflectivities are set to unequal values. Indeed, the factor $r_n^2(1-r_n)^{N-2n}$ for each n is maximized for

$$r_n = \frac{2}{N-2(n-1)}, \tag{12}$$

which corresponds to the fraction of the optical energy reflected towards each pair of detectors being the same. In this case, Eq. (10) transforms into

$$C(r_n) = \frac{N!}{N^N} \approx \sqrt{2\pi N} e^{-N},$$

where we have used the Stirling approximation. In this way, we find the optimal N00N state heralding probability in the limit of low squeezing as

$$\mathrm{pr} \approx \sqrt{8\pi N} e^{-N} \gamma^{2N} \tau^{N/2}. \tag{13}$$

The corresponding numerical data in Supplementary Figure 3 are shown by dashed green lines.

In Supplementary Figure 4, we verify that the N00N state preparation fidelity does not fall below the value predicted by Eq. (8). For high losses ($\tau \ll 1$), that equation predicts the behavior of the fidelity quite precisely up to the second order in γ. For low losses, on the other hand, the fidelity is slightly above that prediction.

Some improvement of preparation probabilities can be gained by using number-discriminating SPCMs. The advantage of these detectors is that they are capable of eliminating the contributions of higher order in γ to the output state, such as the second and third terms in Eq. (8). As a result, one can increase the down-conversion amplitude γ without the risk of degrading the fidelity. However, this advantage is absent if the losses are high, as in that case an N-photon heralding event may occur in response to more than N photons emitted by one of the crystals. This behavior is made evident by the dotted red curves in Supplementary Figure 3.

We estimated the maximum distance at which the N00N states can be prepared at reasonable rates by determining, from the plots in Supplementary Figure 3, the losses corresponding to the N00N state production probabilities of pr $= 10^{-9}$. These values are 58.6, 9.8 and 1.8 dB for the 2-, 4-, and 6-photon N00N states, respectively. Assuming that a fiber channel with a loss rate of 0.2 dB/km is used, these values correspond, respectively, to the distances of 293, 49 and 9 km.

SUPPLEMENTARY REFERENCES

[1] U. Leonhardt, *Measuring the quantum state if light* (Cambridge University Press, 1997).

[2] Kok, P., Lee, H. & Dowling, J. P. Creation of large-photon-number path entanglement conditioned on photodetection. *Phys. Rev. A* **65**, 052104 (2002).

[3] Berry, D. W. & Lvovsky, A. I. Linear-Optical Processing Cannot Increase Photon Efficiency. *Phys. Rev. Lett.* **105**, 203601 (2010).
